# Hybrid Detergents Facilitate Scalable Charge Reduction and Stabilize Membrane Proteins While Retaining Lipid Interactions

**DOI:** 10.1002/chem.202503191

**Published:** 2025-11-12

**Authors:** L.H. Urner, D. Shutin, M.T. Agasid, C.V. Robinson

**Affiliations:** ^1^ Department of Chemistry and Chemical Biology TU Dortmund University Otto‐Hahn‐Str. 6 44227 Dortmund Germany; ^2^ Kavli Institute for Nanoscience Discovery University of Oxford South Parks Road Oxford Ox1 3QU UK

**Keywords:** charge, detergent, lipid, membrane, protein

## Abstract

Membrane protein complexes regulate many biological functions, yet they are incredibly challenging to handle. Detergents are a key enabling technology for purification and analysis as they can provide membrane proteins with controlled solubility, stability, and heterogeneity. However, the design criteria for detergents that enable both the purification and analysis of membrane proteins with their associated lipids remain unclear. Here, we leverage the modular nature of nonionic hybrid detergents to chemically tune molecular parameters that are important for maintaining native oligomeric states of membrane protein complexes and copurifying lipids. Tuning the hydrophilic‐lipophilic balance (HLB), and the relative size of a nonionic head in proximity to a tetraethylene glycol headgroup, led to altered charge reduction, retention of protein oligomers and changed the relative degree of copurifying lipids from purification to analysis in a predictable manner. Our findings represent a significant step forward in the investigation of membrane proteins and interactions with lipids.

## Graphical abstract

Hybrid detergents enable the purification and native mass spectrometry analysis of membrane proteins and associated lipids. Chemical tuning of headgroups in hybrid detergents enables control over charge reduction, oligomer stability, and lipid retention, which enables the analysis of intact membrane proteins and their interactions with associated lipids.

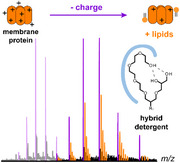

## 1 Introduction

Membrane proteins are important targets for approx. 60% of current drugs.^[^ ^1^, ^2^ ^]^ Noncovalent interactions between proteins and ligands define structures of protein complexes in membranes. Given the importance of these interactions in biochemistry and medical research, native mass spectrometry (nMS) is increasingly used for the relative quantification of how ligands affect structural organization, including proteins, drugs, nucleotides, and lipids.^[^ ^3^, ^4^, ^5^, ^6^, ^7^ ^]^

Despite their ubiquitous importance in daily life, detergents are key reagents for the structural analysis of membrane proteins.^[^ ^8^, ^9^, ^10^, ^11^, ^12^ ^]^ In nMS, protein complexes are solubilized from membranes with detergents and transferred into the vacuum of a mass spectrometer by nano‐electrospray ionization (nESI).^[^ ^12^, ^13^, ^14^ ^]^ Subsequent detergent removal can liberate intact protein complexes for precise mass analysis.^[^ ^12^, ^13^, ^14^ ^]^ Detergents that enable both the purification and nMS analysis of membrane protein complexes are hard to find.^[^ ^15^, ^16^, ^17^ ^]^ Saccharide detergents, like n‐dodecyl‐β‐D‐maltoside (DDM), often enable the purification of intact protein complexes in solution but require harsh collisional activation conditions necessary for detergent removal in the gas‐phase, which leads to Coulomb‐driven protein unfolding and/or dissociation.^[^ ^16^, ^18^, ^19^ ^]^ To better preserve intact protein structures, their charge states can be reduced.^[^ ^15^, ^20^, ^21^ ^]^ Charge‐reducing reagents,^[^ ^20^, ^22^, ^23^, ^24^ ^]^ like detergents,^[^ ^17^, ^25^, ^26^, ^27^ ^]^ often provide harsh solution conditions and promote protein denaturation during sample preparation, such as in the case of tetraethylene glycol monooctyl ether (C8E4).^[^ ^28^ ^]^ Nondenaturing detergents with charge‐reducing properties are rare but offer significant advantages for the investigation of intact membrane protein complexes.^[^ ^16^, ^17^, ^20^, ^26^, ^29^ ^]^

Attempts to design suitable detergents for protein purification and nMS led to the discovery of a hybrid detergent that combines the headgroups of DDM and C8E4 (Mal + E4), stabilized membrane protein complexes but did not efficiently reduce the charge of membrane protein ions.^[^ ^28^ ^]^ Solution and gas‐phase properties of detergents are determined by the chemistry of polar headgroups, such as the maltoside in DDM (Mal) or tetraethylene gycol in C8E4 (E4).^[^ ^16^ ^]^ During electrospray ionization, charges are distributed between detergent and protein according to proton affinity of the detergent, number of detergent molecules, and surface area of the protein.^[^ ^27^ ^]^ We hypothesized that the E4 head in the hybrid detergent (E4 + Mal) is sterically shielded by the DDM headgroup (Mal) and not capable of chelating and transporting charge away from protein ions during ESI.^[^ ^28^ ^]^

**Figure 1:**
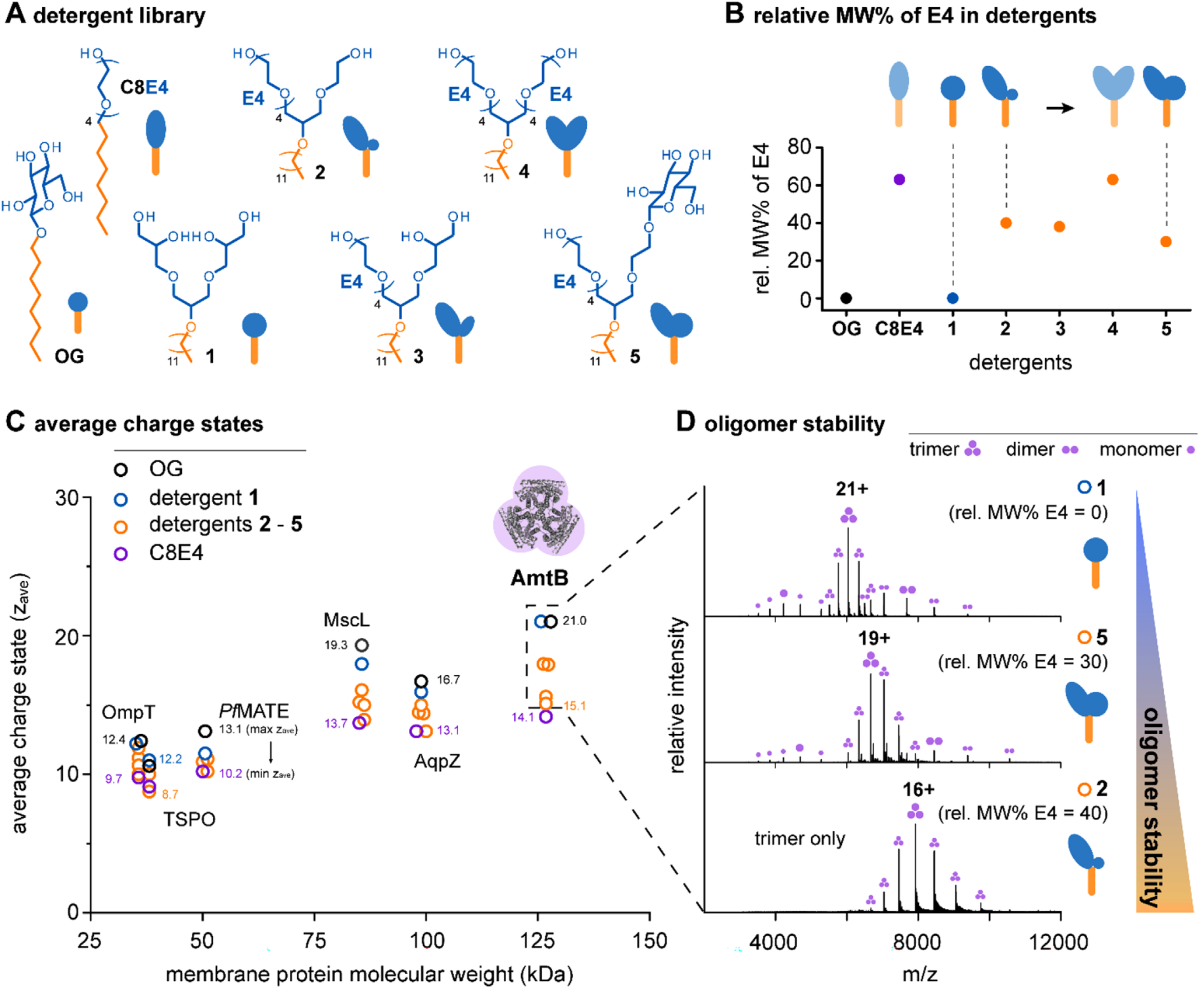
**Detergent headgroups control protein charge states and oligomer stability**. A) Schematic showing structures of detergents used here. B) Diagram showing the relative molecular weight percent (MW%) of E4 in detergent structures. C) Diagram showing average charge states of the largest oligomeric state of each membrane protein complex with different molecular weights obtained upon liberation from different detergents. D) Mass spectra showing signals of AmtB monomers, dimers and/or trimers upon liberation from detergents with increasing relative MW% of E4 in detergent structures.

To gain control over the charge‐reducing properties of detergents, we developed hybrid detergents **2**–**5** which are structurally related to C8E4, n‐octyl‐β‐D‐glucoside (OG), dendritic triglycerol detergent **1** and can enable the purification and nMS analysis of functional proteins (Figure 1A).^[^ ^6^, ^30^ ^]^ The relative size of the headgroups in proximity to E4 in **2**–**5** can be synthetically tuned with unprecedented modularity, which enables predictable control over copurified lipid content and revealed noncanonical protein‐lipopolysaccharide associations in Gram‐negative *E. coli*.^[^ ^6^ ^]^ To find nondenaturing detergents for membrane protein purification and charge reduction, herein, we investigated whether scaling the relative size of the head in proximity to E4 produces hybrid detergents with scalable charge‐reducing properties. Since protein‐lipid binding obtained by nMS can be biased by charge reduction,^[^ ^31^ ^]^ we interrogate whether controlling the relative copurifying phospholipid content with OG, C8E4, and **1**–**5** depends on charge reduction or reflects solution conditions.

To test whether scaling the relative size of the headgroup in proximity to E4 in our hybrid detergents results in predictable scalable charge reduction, we established a protein analysis pipeline composed of six different membrane proteins covering molecular masses from 35 to 130 kDa and oligomeric states from monomers up to pentamers, that is, outer membrane protease T (OmpT), translocator protein (*Rs*TSPO), multiantimicrobial extrusion protein (*Pf*MATE), mechano‐sensitive channel (MscL), water channel (AqpZ), and ammonia channel (AmtB) (Figure 1C). We purified the proteins under mildly delipidating conditions with DDM and exchanged the detergent environment to OG, C8E4, and **1**–**5** using size‐exclusion chromatography. Subsequent nMS analysis under comparable instrument conditions informed on the average charge state (z_ave_) values of intact membrane protein complexes.

To analyze how scaling the relative size of the headgroup in proximity to E4 affects membrane protein charge reduction, we compared the relative molecular weight percent of E4 in detergent structures with z_ave_ values obtained from 42 membrane protein‐detergent combinations (Figure 1B–D). Detergents lacking E4 yielded consistently higher z_ave_ values than detergents containing E4, which illustrates the role of E4 as a charge‐reducing building block (Table S1 and Figure 1C–D).^[^ ^16^ ^]^ Focusing on AmtB, its z_ave_ values, and oligomer stabilities scaled with the relative molecular weight percent of E4 in detergent structures (Figure 1D). We observed similar results, regardless of the protein, as demonstrated by AqpZ (Figure S1) and other membrane proteins (Table S1). Increasing the relative molecular weight percent of E4 to 40% by reducing the size of neighboring nonionic headgroups led to a decrease in sterically shielding of E4, enhanced protein charge reduction, and increased gas‐phase stabilities of membrane protein oligomers (Figure 1D). Hence, mass spectra of membrane proteins liberated from detergent **2** and C8E4 looked similar in terms of charge state distributions, as shown exemplary for trimeric AmtB (Figure S2). Our findings do not preclude the possibility to detect intact oligomeric states with noncharge reducing detergents,^[^ ^16^ ^]^ but clarify that augmenting detergent design to minimize steric shielding of the E4 headgroup in hybrid detergents is a complementary design modality to simultaneously reduce the charge of membrane proteins, minimize collisional activation and preserve noncovalent interactions in membrane proteins during purification and nMS analysis (Figure 1D).^[^ ^16^, ^17^, ^25^, ^26^, ^27^ ^]^

**Figure 2:**
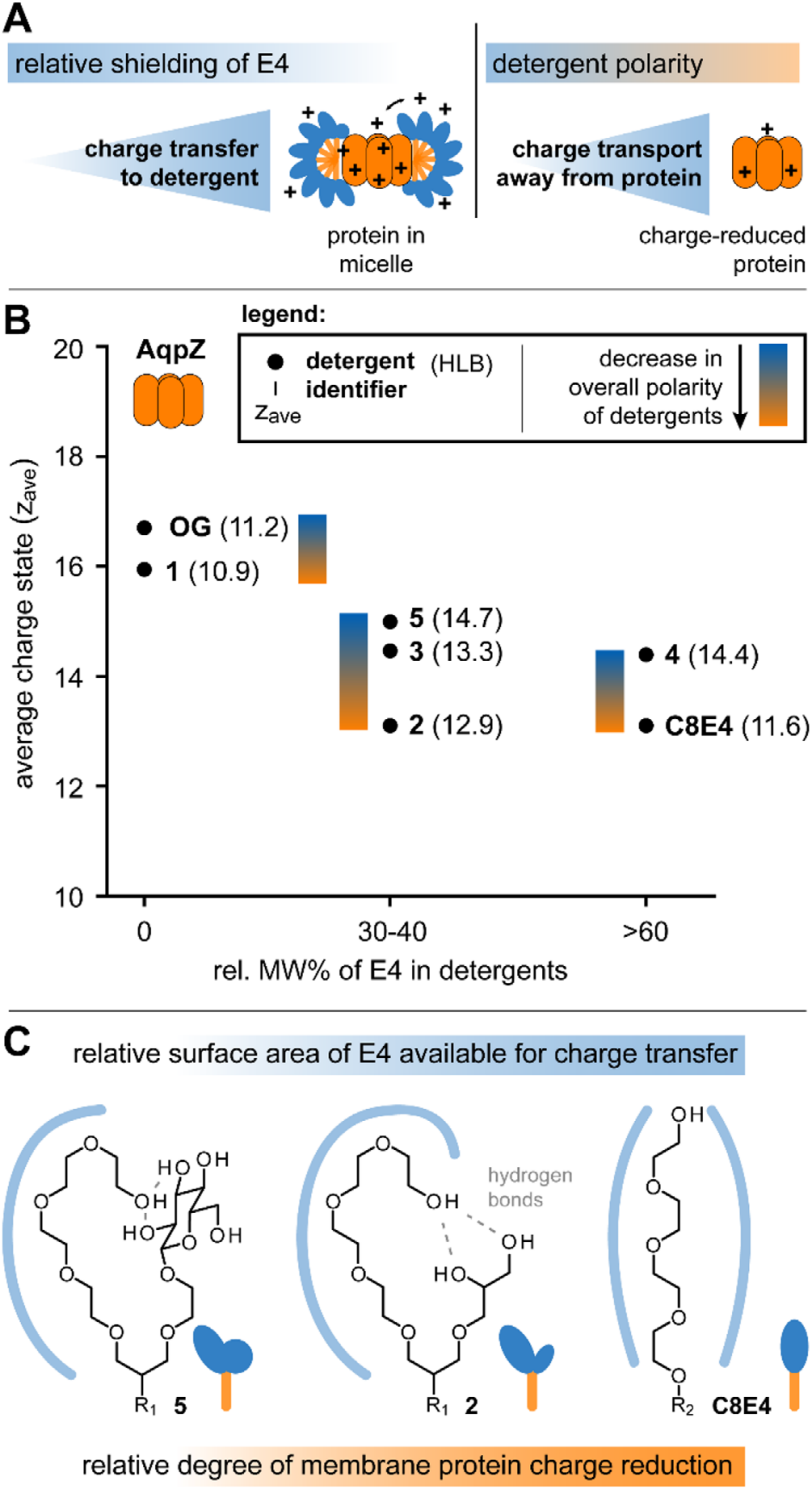
**Polarity and sterically shielding affect protein charge reduction**. A) Schematic indicating how charge reduction correlates with relative shielding of E4 in detergent headgroups, polarity, charge transfer, and charge transport away from the protein. B) Diagram showing average charge states of AqpZ after release from detergents with different relative molecular weight percent of E4 (rel. MW% of E4). Detergent abbreviations are shown with HLB values and relative changes in detergent polarities are highlighted with triangular scalebars. C) Schematic showing the correlation between molecular structure of detergent headgroups, relative surface area of E4 available for charge transfer, and relative degree of membrane protein charge reduction.

How does reducing the size of nonionic headgroups alter membrane protein charge reduction? C8E4 attaches to the hydrophobic surfaces of proteins (Figure 2A). The E4 headgroup can complex cations.^[^ ^16^, ^32^ ^]^ During collision‐induced micelle dissociation, detergents can transport cations away from protein surfaces.^[^ ^27^ ^]^ Charge transfer from proteins to detergents and charge transport away from proteins by dissociating detergents, are key events in charge reduction (Figure 2A).^[^ ^16^, ^27^, ^33^ ^]^

The sizes of the headgroups in our detergents gradually increased from OG to **5** and so does their overall polarity. This led us to the hypothesis that the overall polarity of our detergents affected protein charge reduction (Figure 2A). To investigate the role of detergent polarity, we compared hydrophilic‐lipophilic balance (HLB) values and z_ave_ values obtained with detergents of comparable relative molecular weight percentages of E4, that is, category 1 (0% E4), 2 (30–40% E4), and 3 (>60% E4) (Figure 2B).  HLB values are a metric for polarity, which we calculated from the detergent structures using Griffin's equation.^[^ ^34^ ^]^ The higher the HLB value, the more polar the detergent (Table S2 and Figure 2B). As shown for AqpZ and AmtB, a reduction of z_ave_ values correlated with reduced HLB values (Figure 2B and Figure S3). This indicated decreasing the polarity of detergents enhanced charge reduction possibly by improving the charge transport from membrane proteins (Figure 2A, B).

Complementary to overall detergent polarity, we hypothesized that hydrogen bonding between neighboring headgroups in E4‐based hybrid detergents can impede a charge transfer to the E4 headgroup, thereby affecting protein charge reduction. Gas‐phase infrared spectroscopy studies on our hybrid detergents confirmed that the E4 headgroup can undergo hydrogen bonding with neighboring nonionic groups (Figure S4).^[^ ^35^ ^]^ As the sizes of nonionic headgroups in proximity to E4 is increased, such as in the cases of **2** and **5**, the relative surface area of the E4 head available for charge transfer to the detergent is reduced (Figure 2A and Figure S4). Our findings indicate that increasing the relative surface area of E4 available for charge transfer increases the relative degree of protein charge reduction (Figure 2C).

Membrane protein charge reduction also depends on the number of detergent molecules in ESI droplets.^[^ ^27^ ^]^ This led us to the question of whether our differences in charge reduction can be explained by detergent concentrations. For example, E4‐containing detergents could lower the charge of proteins depending on the number of detergent molecules within nESI droplets, which is determined by the detergent concentration in nMS buffer, that is, two times the critical aggregation concentration (2x cac).^[^ ^16^ ^]^ Detergent concentrations are routinely adjusted to 2x cmc to secure membrane protein solubility and stability in assay buffers.^[^ ^28^, ^36^ ^]^ However, the cac of the E4‐containing hybrid detergent **2** (1 mM) was eight times lower compared to C8E4 (8 mM). Both detergents exhibited comparable charge‐reducing properties, regardless of the membrane protein (Table S1 and Figure 2B). Hybrid detergents **2**–**5** required more similar concentrations during purification and analysis (0.8–2 mM) but produced different z_ave_ values (Figure 2B). We expect membrane protein charge reduction in these detergents **2** ‐ **5** is determined by the ability of E4 to capture charge, overall polarity and H‐bonding between headgroups.

Controlling steric shielding of the E4 headgroup and polarity of hybrid detergents are design modalities to control charge reduction. Recent nMS experiments demonstrated that detergents OG, C8E4, **1**–**5** enabled gradual control over the relative degree to which proteins copurify with lipids.^[^ ^6^ ^]^ Protein‐lipid binding obtained by nMS can be biased by charge reduction.^[^ ^31^ ^]^ This led us to the question of whether protein charge reduction determined the relative degree to which proteins were detected in complex with copurified lipids by nMS. The ability to control the retention of protein‐lipid interactions is critically important for the investigation of protein‐lipid interactions in reconstituted membranes and protein function.^[^ ^11^, ^37^, ^38^, ^39^ ^]^ Mildly delipidating detergents enable the identification of copurifying lipids via omics techniques.^[^ ^11^, ^39^ ^]^ Strongly delipidating detergents provide clean proteins to which lipids are exogenously added back for structure‐function and activity studies.^[^ ^11^, ^37^, ^38^ ^]^ A better understanding of how detergents drive protein delipidation is critically important to support the investigation of lipids in protein structure and function.^[^ ^6^, ^11^, ^12^, ^17^, ^40^, ^41^ ^]^ The question of whether lipid binding obtained upon protein purification with C8E4, OG, **1**–**5** by nMS depends on charge reduction or reflects solution conditions has not been fully addressed, yet.^[^ ^6^ ^]^

**Figure 3:**
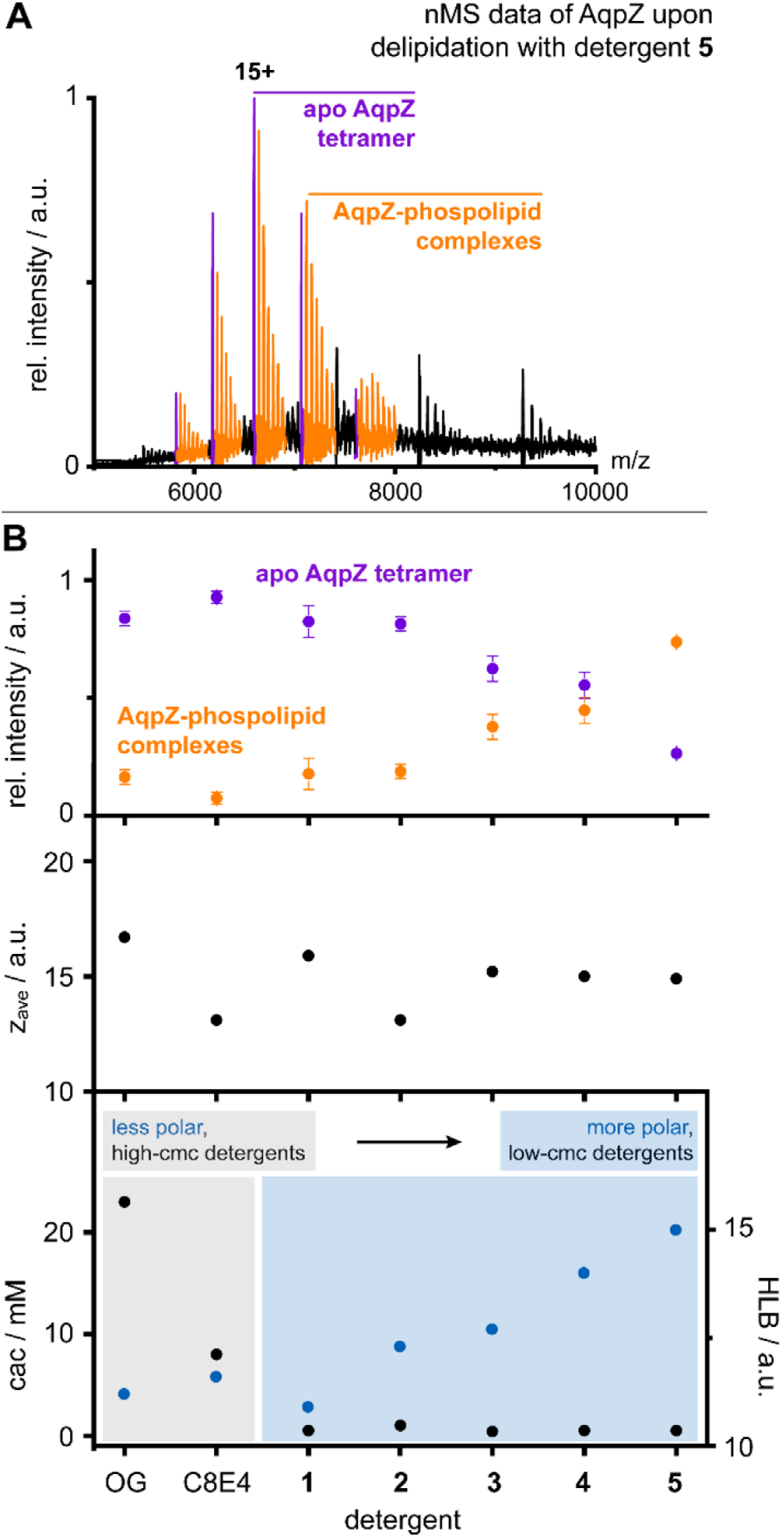
**Detergent properties determine membrane protein delipidation**. A) nMS spectrum showing signals of the apo state of tetrameric AqpZ and lipid‐bound states obtained upon delipidation with hybrid detergent **5**. B) Diagrams showing the relative intensities of apo form and protein‐lipid complexes of AqpZ (±SEM, n = 3) (upper pannel); average charge states of AqpZ (middle pannel); and properties of detergents that were used for AqpZ delipidation, including cac and HLB values (lower pannel).

To understand whether trends in delipidation observed by nMS with OG, C8E4, **1**–**5** reflect solution or gas‐phase conditions, we investigated all samples using comparable instrument conditions. To investigate whether detergent concentration and/or charge reduction determine the relative intensity of membrane protein‐lipid complexes, we compared relative intensities of free proteins and protein‐lipid complexes with detergent concentrations and z_ave_ values. We observed almost quantitative delipidation for C8E4 and OG, which have opposing charge‐reducing properties, but also cac values that are higher (8–23 mM) compared to the other detergents **1**–**5** (0.4–1 mM) (Table S3 and Figure 3B). We concluded that the strong delipidating properties of C8E4 and OG are primarily determined by their concentration. This agrees with the idea that proteins and their lipid complexes in solution are in an equilibrium that can be shifted toward delipidated proteins when the detergent concentration is increased.^[^ ^9^, ^28^, ^42^ ^]^ Detergent concentrations in purification buffers are traditionally adjusted to 2x cac.^[^ ^28^, ^36^ ^]^ Therefore, detergents with higher cac values, like OG and C8E4, exhibit stronger delipidating properties than detergents with lower cac values (Figure 3B). A limitation of this argument is that neither charge reduction or detergent concentration can explain the gradual changes in delipidation that we subsequently observed among the detergents **1**–**5** (Figure 3B).

Since the concentrations used of the detergents **1**–**5** was in a comparable range, we asked whether changes in detergent polarity could explain our delipidation outcomes (Table S3 and Figure 3B). Detergents compete with protein‐lipid binding via hydrophobic and hydrophilic interactions.^[^ ^8^, ^9^ ^]^ The lower the overall polarity of a detergent, the less effective a detergent would be for delipidation. In line with this explanation, as a function of detergent HLB, we observed a gradual increase in relative intensities of lipid complexes, along with a gradual decrease in the *apo* form (Figure 3B). Tuning the hydrophobicity of micellar aggregates with similar cac values is a complementary modality to control the relative degree to which proteins copurify with lipids (Figure 3B). The charge‐reducing gold standard detergent for nMS studies on membrane proteins is C8E4.^[^ ^14^, ^16^, ^26^, ^28^, ^37^ ^]^ This detergent produced the lowest charge states observed for membrane proteins, has a high cac, which typically leads to delipidation and protein denaturation (Figure 3A, B). Compared to C8E4, our hybrid detergents have lower cac values and enable membrane protein charge reduction and a better retention of copurifying lipids (Figure 3A, B). As a result of these properties, hybrid detergents will facilitate the purification and analysis of membrane protein‐lipid interactions.

Seen from a broader perspective, our detergents add to the growing repertoire of reagents that can be used to manipulate the charge of proteins in the gas phase. Many reagents studied today reduce the charge of membrane proteins by exhibiting functional groups with high gas‐phase basicity values.^[^ ^17^, ^29^, ^33^, ^43^, ^44^, ^45^ ^]^ Our hybrid detergents are polyether/polyol structures whose functional groups do not exhibit gas‐phase basicity values that are sufficiently high to cause protein charge reduction.^[^ ^33^ ^]^ The ability of hybrid detergents to reduce the charge of membrane proteins results more likely from the stabilization of protons in the detergent headgroup by chelation. The oxygen atoms in the polyether/polyol headgroups can stabilize protonated sides in detergent headgroup in the absence of solvent.^[^ ^46^ ^]^ Future studies focusing on gas‐phase dissociation pathways of membrane protein‐detergent complexes could help to understand whether hybrid detergents reduce the charge of membrane proteins by subtracting protons from proteomicelles during detergent removal and/or competing with charging during the ESI process.^[^ ^16^, ^19^, ^33^ ^]^

The final question that has not yet been fully addressed in our discussion is whether the relative degree in the retention of protein‐lipid complexes observed by nMS is a solution phenomenon.^[^ ^17^ ^]^ In solution, detergent micelles containing membrane protein‐lipid complexes are in equilibrium with micelles containing proteins or lipids. Since detergents and lipids compete for binding to proteins, adding more detergent to a membrane protein preparation can shift the equilibrium in solution from micelles containing protein‐lipid complexes toward micelles containing proteins or lipids.^[^ ^42^ ^]^ This can explain the strong delipidating properties of high‐cmc detergents OG and C8E4 compared to our low‐cmc hybrid detergents **1**–**5** (Figure 3B).^[^ ^28^ ^]^ The spectra obtained from membrane proteins analyzed in combinations with **1**–**5** were acquired using comparable protein and detergent concentrations (Figure 3B). To clarify whether the relative retention of protein‐lipid complexes is a solution phenomenon or due to different gas‐phase stabilities, we compared relative intensities of protein‐lipid complexes with z_ave_ values observed upon detergent removal (Figure 3B). Charge‐reducing reagents lower Coulomb‐repulsive interactions during the detergent removal process, which can increase relative intensities of protein‐lipid complexes observable by nMS.^[^ ^31^ ^]^ Controversially, our hybrid detergent **2** led to lower z_ave_ values and lower relative intensities of protein‐lipid complexes, while our hybrid detergents **3**–**5** led to higher z_ave_ values and higher relative intensities of protein‐lipid complexes (Figure 3B). Accordingly, membrane protein‐lipid complexes liberated from hybrid detergents **3**–**5** experienced harsher activation conditions in nMS than in the case of **2** under our employed experimental conditions. Hybrid detergents **3**–**5** led to a better retention of protein‐lipid‐complexes than **2** (Figure 3B). This led us to the conclusion that relative intensities of protein‐lipid complexes depend more on the stabilization in solution rather than on an electrostatic activation bias the gas‐phase.^[^ ^17^ ^]^ It is assumed that more hydrophobic detergent aggregates interfere more efficiently with lipid binding in solution than less hydrophobic detergent aggregates.^[^ ^6^ ^]^ This agrees with the observation that the relative intensity of protein‐lipid complexes increases with the increase in HLB of our detergents **1**–**5** (Figure 3B).^[^ ^6^ ^]^

In summary, current purification protocols rely on numerous parameters, which complicate the rational design and assessment of detergents. This makes it challenging to determine how cross‐functional detergents can effectively maintain the structure and function of membrane protein complexes during purification and downstream analysis. The hybrid detergents introduced here allow for predictable control over membrane protein charge reduction, oligomer stability, and lipid retention through chemical tuning of the HLB and steric shielding of the headgroups. Interestingly, nMS data can be obtained for protein concentrations as low as 100 nM∙L^−1^, as shown exemplary for trimeric AcrB in **1** (Figure S5), which emphasizes the utility of our detergents for nMS applications. Compared to C8E4, our detergents offer tuneable delipidating and charge‐reducing properties, which enables either the identification of copurifying lipids or adding lipids back to delipidated proteins.^[^ ^11^ ^]^ Our findings deliver detergents that support the analysis of regulatory roles that lipids can have in membrane protein structure and function.

## Supporting Information

The authors have cited an additional reference within the Supporting Information.^[^ ^47^ ^]^

## Conflict of Interest

The author declares no conflict of interest.

## Supporting information



Supporting Information

## Data Availability

The data that support the findings of this study are available in the supplementary material of this article.
