# Supplementary material for: Hybrid Detergents Facilitate Scalable Charge Reduction and Stabilize Membrane Proteins While Retaining Lipid Interactions
Source: Chemistry. 2025 Nov 12;31(72):e03191. doi: 10.1002/chem.202503191 (PMC12731538; doi:10.1002/chem.202503191)
Supplement: Supplementary file 1 — Supporting Information [file CHEM-31-e03191-s001.docx]

**Table of contents**

[**Supplementary Tables 2**](#_Toc213069219)

[**Table S2. Hydrophilic-lipophilic balance values of detergents. 3**](#_Toc213069220)

[**Table S3. Critical aggregation concentration and utilized detergent concentrations. 4**](#_Toc213069221)

[**Supplementary Figures 5**](#_Toc213069222)

[**Figure S1: Hybrid detergents increase oligomer stability of AqpZ. 5**](#_Toc213069223)

[**Figure S2. Charge state distributions of trimeric AmtB in charge-reducing detergents. 6**](#_Toc213069224)

[**Figure S3: Hybrid detergent polarity affects charge reduction of AmtB. 7**](#_Toc213069225)

[**Figure S4. Computed and experimental infrared spectra and detergent structures. 8**](#_Toc213069226)

[**Figure S5. nMS spectrum of trimeric AcrB. 9**](#_Toc213069227)

[**Materials and methods 10**](#_Toc213069228)

[**Detergents 10**](#_Toc213069229)

[**Hydrophilic-lipophilic balance 10**](#_Toc213069230)

[**Critical aggregation concentration 10**](#_Toc213069231)

[**Membrane preparation for native mass spectrometry 10**](#_Toc213069232)

[**Refolding of OmpT for native mass spectrometry 11**](#_Toc213069233)

[**Membrane protein extraction and affinity purification 12**](#_Toc213069234)

[**Detergent exchange for native mass spectrometry 12**](#_Toc213069235)

[**Native mass spectrometry 13**](#_Toc213069236)

[**Average charge state calculation 13**](#_Toc213069237)

[**Relative intensities of apo form and protein-lipid complexes 13**](#_Toc213069238)

[**References 14**](#_Toc213069239)

# Supplementary Tables

| **T**able S1. Average charge states of membrane protein-detergent combinations. Summary of average charge states (z_ave_) obtained from 42 protein-detergent combinations. |
| --- |
| \|  \|  \| **membrane proteins** \| \| \| \| \| \| \| --- \| --- \| --- \| --- \| --- \| --- \| --- \| --- \| \| **z_ave_** \| **OmpT** \| **TSPO** \| **PfMATE** \| **MscL** \| **AqpZ** \| **AmtB** \| \| **d**  **e**  **t**  **e**  **r**  **g**  **e**  **n**  **t**  **s** \| **OG** \| 12.4 \| 10.6 \| 13.1 \| 19.3 \| 16.7 \| 22.3 \| \| **C8E4** \| 9.7 \| 9.1 \| 10.2 \| 13.7 \| 13.1 \| 15.6 \| \| **1** \| 12.2 \| 11.0 \| 11.5 \| 17.9 \| 15.9 \| 19.2 \| \| **2** \| 10.0 \| 8.7 \| 10.2 \| 13.9 \| 13.1 \| 16.3 \| \| **3** \| 11.1 \| 10.0 \| 10.2 \| 14.4 \| 14.4 \| 16.9 \| \| **4** \| 10.6 \| 10.5 \| 10.9 \| 14.3 \| 14.3 \| 16.4 \| \| **5** \| 11.9 \| 10.0 \| 11.1 \| 16.0 \| 14.9 \| 18.8 \| |

Table S2. Hydrophilic-lipophilic balance values of detergents. Summary of hydrophilic-lipophilic balance (HLB) values obtained from our detergents, including a summary of detergent molecular weights (MW) and the molecular weights of detergent tails (MW_tail_).

| **detergent** | **MW** | **MW_tail_** | **HLB** |
| --- | --- | --- | --- |
| OG | 292 | 129 | 11.2 |
| C8E4 | 306 | 129 | 11.6 |
| 1 | 408 | 185 | 10.9 |
| 2 | 480 | 169 | 12.9 |
| 3 | 510 | 169 | 13.3 |
| 4 | 612 | 169 | 14.4 |
| 5 | 642 | 169 | 14.7 |

Table S3. Critical aggregation concentration and utilized detergent concentrations. Summary of critical aggregate concentration (cac) values of our detergents and detergent concentrations utilized in purification and nMS buffers (2x cac).

| **detergent** | **cac / mM** | **2x cac / mM** |
| --- | --- | --- |
| OG | 23 | 46 |
| C8E4 | 8 | 16 |
| **1** | 0.5 | 1 |
| **2** | 1 | 2 |
| **3** | 0.4 | 0.8 |
| **4** | 0.5 | 1 |
| **5** | 0.5 | 1 |

# Supplementary Figures

**
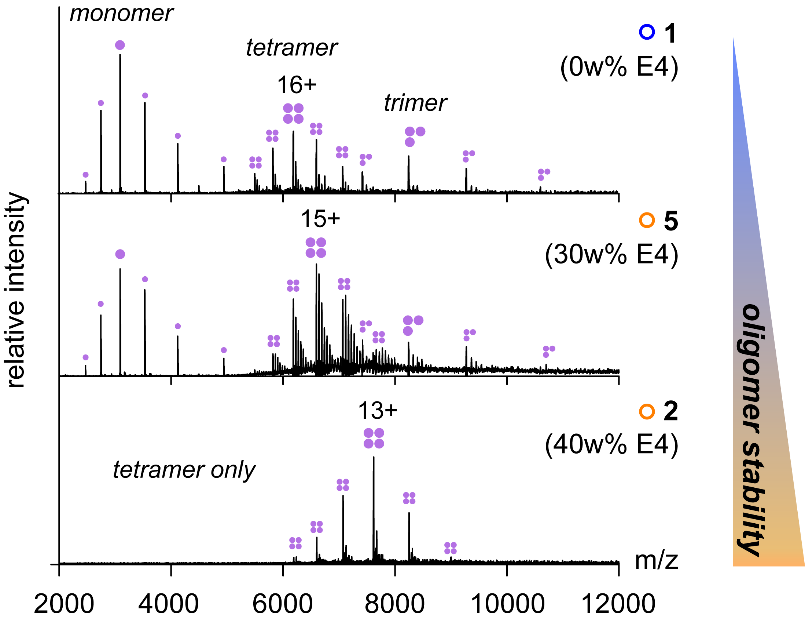
**

Figure S1: Hybrid detergents increase oligomer stability of AqpZ. Mass spectra of AqpZ obtained upon liberation from detergents (**1**, **5**, **2**) with increasing relative MW% of E4 in detergent structures. Mass spectra were obtained using similar instrument conditions.

**
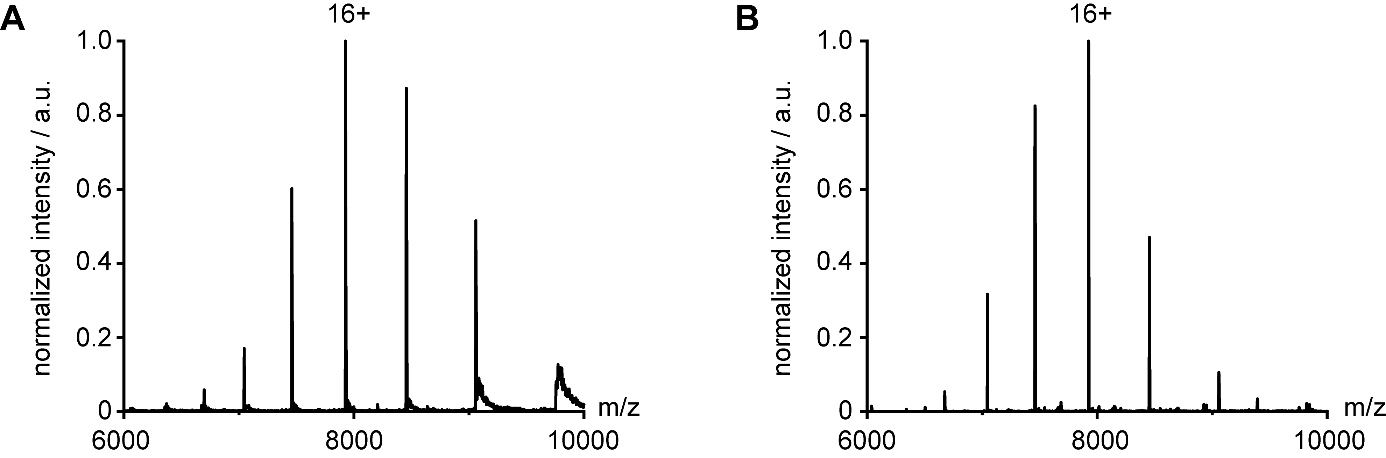
**

Figure S2. Charge state distributions of trimeric AmtB in charge-reducing detergents. Mass spectra of trimeric AmtB liberated from **A** C8E4 and **B** detergent **2**. The most abundant charge state is labelled as 16+.

**
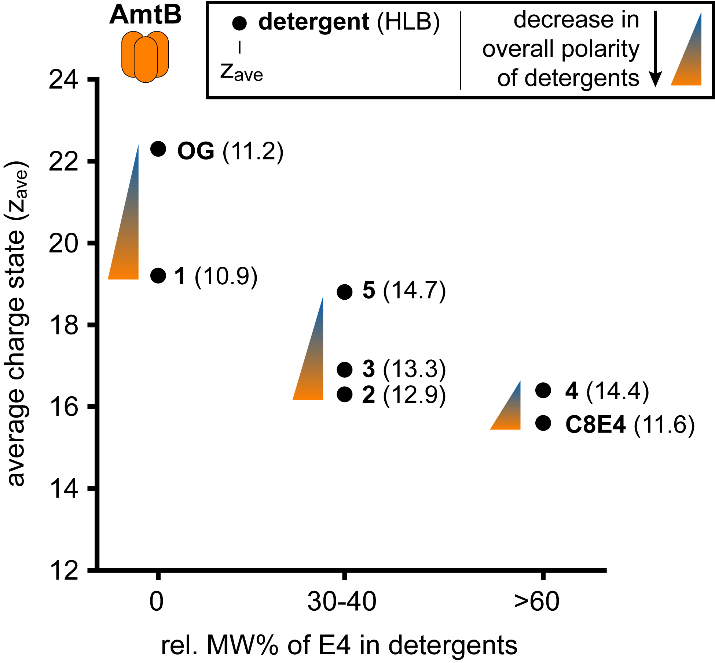
**

Figure S3: Hybrid detergent polarity affects charge reduction of AmtB. Diagram showing average charge states (z_ave_) of AmtB after release from detergents with different relative molecular weight percent of E4 (rel. MW% of E4).


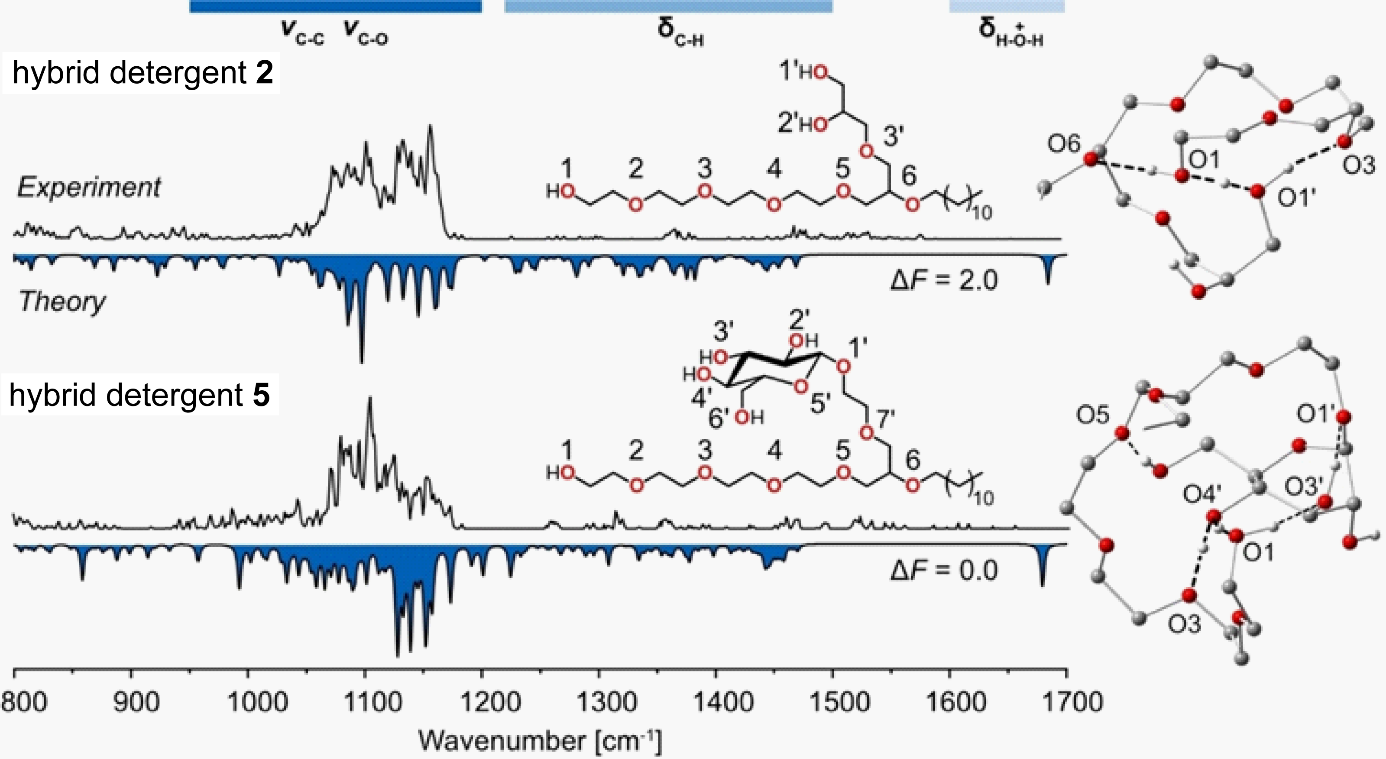


Figure S4. Computed and experimental infrared spectra and detergent structures.[1] Typical regions of stretching (*v*) and bending (*δ*) vibrations observed from singly protonated detergent ions in the spectra are indicated. The core interactions and relevant oxygen atoms are highlighted with hydrocarbon chains truncated and aliphatic hydrogens removed for visibility. The glucose ring of hybrid detergent**5** is shaded for orientation in the computed structure. The computed free energies (Δ*F*) relative to the lowest-energy conformer are indicated in kJ mol^−1^. Structure optimization and frequency analysis were performed at the PBE0+D3/6-311+G(d,p) level of theory and spectra were scaled by a factor of 0.965. Copyright: The image and figure caption were adapted with minor modifications from reference [1] with permission from the authors (CC BY 4.0).

**
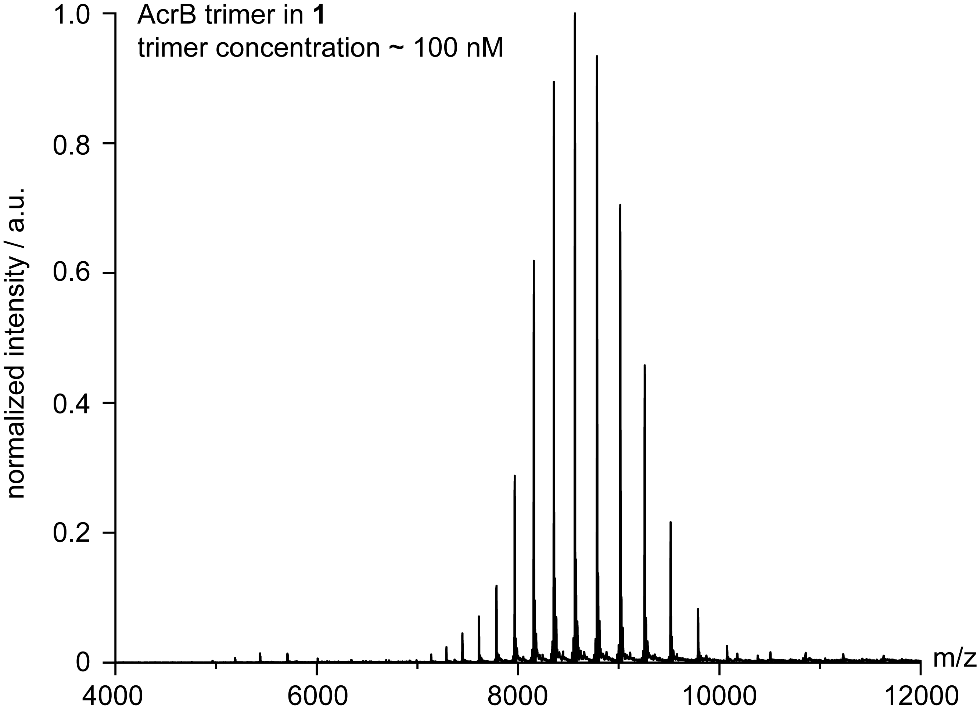
**

Figure S5. nMS spectrum of trimeric AcrB. Diagram showing a mass spectrum of trimeric AcrB in detergent **1** at a trimer concentration of 100 nM in solution.

# Materials and methods

## Detergents

The hybrid detergents **1**-**5** were synthesized as described in detail in Ref.^[2]^ The detergents C8E4 and OG were purchased from Glycon – Biochemicals GmbH and used as supplied.

## Hydrophilic-lipophilic balance

HLB values were determined as described before: The HLB values of OG, C8E4, **1**-**5** were calculated using the well-known expression:

$HLB=20*\left( 1-\frac{MW_{tail}}{MW} \right)$,

in which individual parameters are defined as follows: molecular weight of the tail (MWtail) and molecular weight of the detergent (MW). HLB values are summarized in Table S2.

## Critical aggregation concentration

Critical aggregation concentration (cac) values of detergents were determined using dynamic light scattering (DLS) by following a previously published procedure:^[3]^ Serial dilutions with detergent concentrations between 10^−8^ and 10^−2^ M were prepared in MilliQ water. The samples were filtered (RC, 0.2 µm) and equilibrated for 16 h at room temperature before the analysis. The samples were transferred into a quartz cuvette (Quartz Suprasil, width × length: 2 mm × 10 mm) and analyzed with a Zetasizer Nano-ZS ZEN3600 (Malvern, UK). The instrument was operated with the Zetasizer Software (v7.11) and the following acquisition parameters were used: material (polystyrene latex), dispersant (water), sample viscosity parameters (use dispersant viscosity as sample viscosity), temperature (22.5 °C), equilibration time (120 s), cell type (quartz cuvettes), measurement angle (173° backscatter), measurement duration (manual), number of runs (11), run duration (10 s) number of measurements (3), delay between the measurements (0 s), data processing (general purpose, normal resolution). The derived count rate values obtained from three measurements (per concentration) were averaged and the logarithm of the derived count rate average was plotted against the logarithm of the detergent concentration. The double logarithmic plots showed two characteristic regions: (a) a flat region with low count rates at lower OGD concentrations and (b) a linear growth in the derived count rate at higher OGD concentrations. The individual regions were fitted to linear functions and the cac value was calculated from their intersection. The cac values of all detergents are summarized in Table S3.

## Membrane preparation for native mass spectrometry

Protein-containing membranes for protein purification and nMS analysis of *Pf*MATE, MscL, AmtB, and AqpZ were prepared using an established procedure:^[4]^ *Pf*MATE-GFP, MscL-GFP, MBP-AmtB, and AqpZ-GFP were expressed by using the following procedure: Plasmids were transformed into C43 (DE3) cells (purchased from Cambridge Bioscience) by mixing 1 μL of plasmid solution (plasmid concentration = 100 ng/μL) with a 25 μL aliquot of C43 cells. The cells were incubated on ice for 30 min, heat shocked at 42 °C for 45 s, and cooled on ice for 2 min. LB Broth (450 μL of a 25 g/L aqueous solution) was added. The mixture was shaken with 180 rpm at 37 °C for 1 h. One 50 μL aliquot of this mixture was plated on an agar plate (agar medium composition: 25 g/L LB Broth and 15 g/L agar in water, supplemented with 100 μg/mL ampicillin). The plate was stored overnight at 37 °C. Up to five colonies were picked and transferred into starter culture medium (5 mL of 25 g/L LB Broth, supplemented with 100 μg/mL of ampicillin). The mixture was shaken with 180 rpm at 37 °C for 8 h. The starter culture was transferred into overnight culture medium (400 mL of 25 g/L LB Broth, supplemented with 100 μg/mL of ampicillin). The mixture was shaken with 180 rpm for 15 h at 37 °C. The overnight culture was transferred into in 12 L expression medium (12 × 1 L of 25 g/L LB Broth, supplemented with 100 μg/mL of ampicillin). Cells were shaken with 180 rpm at 37 °C until an optical density value at 600 nm (OD_600_) between 0.7 and 1.0 was reached. Protein expression was induced by adding isopropyl-ß-D-thiogalactopyranoside (12 × 1 mL of a 0.5 M aqueous solution). The cells were shaken with 180 rpm at 37 °C for four hours. Cells were harvested by centrifugation (5,000 × g, 10 min), suspended in 100 mL suspension buffer (20 mM Tris, 300 mM NaCl, 20% v/v glycerol, pH=7.4, supplemented with two cOmplete^TM^ EDTA-free protease inhibitor tablets (Roche)), and lysed using a Microfluidizer. After supernatant clarification (20,000 × g, 20 min, 4 °C), the membranes were pelleted down (125,000 × g, 45 min, 4 °C), resuspended in 6 mL suspension buffer, and then homogenized. The membrane suspension was separated into 2 mL aliquots, which were frozen in liquid nitrogen and stored at - 80 °C for up to two years.

TSPO was expressed by autoinduction using the following procedure:^[5]^ The plasmid for the translocator protein from *Rhodobacter sphaeroides* (*Rs*TSPO) was transformed into BL21 (DE3) *E. coli* cells (Cambridge Bioscience) by mixing 1 μL of plasmid solution (100 ng/μL) with a 50 μL aliquot of the cells^[5]^. The mixture was incubated on ice for 30 min, heat shocked at 42 ^o^C for 45 s, and cooled on ice for 2 min. LB Broth (450 µL of a 25 g/L aqueous solution) was added and the mixture was shaken at 37 °C with 180 rpm for 1 h. One 50 µL aliquot of this mixture was plated on an agar plate (agar medium composition: 25 g/L LB Broth and 15 g/L agar in water, 50 µg/mL kanamycin) and the plate was incubated overnight at 37 °C. Six colonies were transferred to 200 mL of LB medium containing 50 µg/mL kanamycin. The so-obtained mixture (overnight culture) was shaken at 30 °C with 180 rpm for 14 h. Autoinduction medium ZYM-5052 supplemented with 100 µg/mL kanamycin was prepared (12 x 1 L portions). Every 1 L portion of autoinduction medium was supplemented with 15 mL overnight culture. The mixtures were shaken at 30 °C with 220 rpm until the optical density at 600 nm exceeded a value of four (OD_600_ > 4). The cells were harvested by centrifugation (5,000 x *g*, 10 min), the supernatant was discarded, and the harvested cells were resuspended in 100 ml lysis buffer (50 mM KH_2_PO_4,_ 1 mM EDTA, pH = 6.5, two cOmplete^TM^ EDTA-free protease inhibitor tablets (Roche)). Cells were lysed using a Microfluidizer. The supernatant was clarified by centrifugation (20,000 × g, 20 min, 4 °C). The supernatant was isolated and subjected to ultracentrifugation (125,000 × g, 1 h, 4 °C). The supernatant was discarded, and the membrane pellet was homogenized with 10 mL suspension buffer (20 mM Tris, 100 mM NaCl, 20% w/v glycerol, pH = 7.4, one cOmplete^TM^ EDTA-free protease inhibitor tablet per 50 mL of buffer). The membrane suspension was separated into 2 mL aliquots, flash frozen with liquid nitrogen, and stored for up to two years at ‑80 °C.

## Refolding of OmpT for native mass spectrometry

OmpT was isolated from *E. coli* exclusion bodies and refolded to make it ready for detergent exchange using the following established procedure:^[3]^ The plasmid encoding the G236K/K237G mutant of OmpT was expressed in BL21 cells (DE3, purchased from New England Biolabs) in 12 L batches (12 × 1 L LB Broth, supplemented with 50 µg/mL kanamycin) as described above.^[6]^ Cells were harvested (5000*×g*, 10 min), suspended in 30 mL suspension buffer (20 mM Tris, 150 mM NaCl, pH = 7.6, protease inhibitor), and lysed using a Microfluidizer. The insoluble material was spun down (5000 × *g*, 10 min, 4 °C), the supernatant was discarded, the pellet was suspended in 30 mL suspension buffer, and the insoluble material was spun down again. The supernatant was discarded and the insoluble inclusion bodies obtained from 12 L culture were suspended in buffer (50 mL of 20 mM Tris, 150 mM NaCl, 8 M urea, pH = 7.6, protease inhibitor) and stirred for 1 h at room temperature. The supernatant was clarified by centrifugation (20000 × *g*, 20 min). An aliquot of the so-obtained urea solution (1 mL) was mixed with refolding buffer (8 mL of 20 mM Tris, 150 mM NaCl, pH = 7.6, and 0.57 w% detergent) and agitated over night at 4 °C. The supernatant was clarified by centrifugation (4000 g, 30 min) and IMAC columns were prepared as described above using glycerol-free buffers. The columns were loaded with clarified supernatants and washed with IMAC wash buffer (2.5 mL of 50 mM Tris, 200 mM NaCl, 20 mM imidazole, pH = 8). OmpT was eluted with IMAC elution buffer (550 µL of 50 mM Tris, 200 mM NaCl, 250 mM imidazole, pH = 8). For the enrichment of folded OmpT, the freshly eluted protein solutions were exchanged into IMAC wash buffer using a desalting column (column volume = 5 mL, GE Healthcare, product number: GE29-0486-84). So-obtained protein solutions were loaded onto freshly prepared IMAC columns and washed with IMAC wash buffer (1 mL). OmpT was eluted again using an intermediate imidazole concentration (1.5 mL of 50 mM Tris, 200 mM NaCl, 40 mM imidazole, pH = 8). The protein was concentrated in a centrifugal filter (MWCO = 30 kDa) to a final concentration of 15 µM and stored at -80 °C.

## Membrane protein extraction and affinity purification

Membrane protein extraction and purification was done using a previously established procedure:^[4]^ One membrane aliquot (2 mL) was added to a mixture of 9 mL buffer (20 mM Tris, 100 mM NaCl, 20% w/v glycerol, pH = 7.4, one cOmplete^TM^ EDTA-free protease inhibitor tablet per 50 mL of buffer) and 1 mL DDM stock solution (10w% w/v DDM in MilliQ water). The suspension was gently agitated for one hour at 4 °C. The supernatant was clarified by centrifugation (4,000 × g, 30 min, 4 °C). IMAC purification was performed as follows: 7 mL of nickel-nitrilotriacetic acid (Ni-NTA) agarose suspension (50%, Qiagen) was mixed with 23 mL of deionized water in a 50 mL Falcon tube. The supernatant was clarified by centrifugation (4,000 × g, 2 min, 4 °C), discarded, and the procedure was repeated. Ni-NTA resin was suspended with 5 mL IMAC wash buffer (50 mM Tris, 200 mM NaCl, 20 mM imidazole, 10% w/v glycerol, 2x cac of DDM, pH = 8) and the protein-containing supernatant was added. The mixture was agitated at 4 °C for 15 minutes and then transferred into an empty gravity flow column (14 cm high, 1.5 x 1.2 cm polypropylene column, purchased from Bio-Rad). Unbound protein was allowed to flow through. The IMAC resin was washed with 45 mL IMAC wash buffer, 45 mL of a IMAC wash/elute buffer mixture (9/1 v/v, 2x cac of DDM, pH = 8), and the protein was eluted with 10 mL IMAC elute buffer (50 mM Tris, 200 mM NaCl, 250 mM imidazole, 10% w/v glycerol, 2x cac of DDM, pH = 8). The eluted membrane protein was concentrated to a final volume of 5 mL with Amicon® filters with molecular weight cut-offs (MWCO) that were adjusted to the molecular weight of the expected membrane protein oligomer embedded in a detergent micelle (MWCO = 50 kDa for TSPO and *Pf*MATE; MWCO = 100 kDa for AqpZ GFP, MscL-GFP, AmtB-MBP).

His-tagged Tobacco Etch Virus (TEV) protease (1-2 mg) was added to remove GFP-His or MBP from individual membrane proteins. The mixture was transferred into dialysis cassettes (MWCO = 3.5 kDa) and dialyzed for 16 h at 4 °C against dialysis buffer (50 mM Tris, 200 mM NaCl, 20 mM imidazole, 10% w/v glycerol, 2x cac of DDM, 1 mM 2‑mercaptoethanol or dithiothreitol, pH = 8). TSPO, AcrB and BtuCD were dialyzed under similar conditions without adding TEV. Subsequently, IMAC columns were washed with 20 mL dialysis buffer. The dialyzed protein solution was passed over the column and the flow-though was collected to remove His-TEV, free GFP-His or His-MBP, and uncleaved membrane proteins. The column was washed with 5 mL dialysis buffer and the flow-through was collected. The combined flowthroughs and wash fractions were concentrated in Amicon® filters until a protein concentration of more than 20 µM per expected protein oligomer was reached. The proteins were purified over a 24 mL SEC column (Superdex 200 Increase 10/300GL column, GE) equilibrated with SEC buffer (50 mM Tris, 150 mM NaCl, 10% w/v glycerol, 2x cac of DDM, pH = 8). Eluted proteins were concentrated in Amicon® filters until a protein concentration of more than 15 µM per expected protein oligomer was reached. The protein solutions were separated into 45 µL aliquots, flash frozen in liquid nitrogen, and stored at -80 °C for up to two years.

## Detergent exchange for native mass spectrometry

All detergent exchange experiments were performed at 4 ^o^C and done by following a previously established procedure:^[4]^ Membrane protein-PL complexes were delipidated by exchanging DDM for a detergent of interest over a 3 mL SEC column (Superdex G-200 Increase 5/150GL column) equilibrated with 200 mM ammonium acetate (pH 6.9) supplemented with the detergent of interest (2x cac). The column was equilibrated with an ÄKTA setup that was operated at 4 °C. The Äkta was equipped with a sample fractionator and the chromatogram was monitored with a UV/VIS detector. The column was washed with 1.2 column volumes (CVs) of deionized water, 1 CV of an aqueous sodium hydroxide solution (0.5 mM), and 1.2 CVs of deionized water before equilibrating with 1.2 CVs of ammonium acetate solution (200 mM, pH = 6.9, 2x cac of the detergent of interest). For every detergent exchange experiment, an aliquot containing the protein of interest (45 µL of a 15 µM solution containing the protein oligomer that was purified before by 24 mL SEC or obtained upon refolding) was injected into the 3 mL SEC column that was equilibrated with the new detergent. The protein was eluted over 1.5 CVs of detergent-containing ammonium acetate solution at a flow rate of 0.2 mL/min. The main protein-containing fractions were pooled and concentrated using Amicon® Ultra 0.5 mL centrifugal filters to a final volume of about 20 to 30 µL. The MWCO cut-off of the Amicon® filters was adjusted to the MW of the target protein (MWCO = 50 kDa for *Rs*TSPO, OmpT, *Pf*MATE; MWCO = 100 kDa for AqpZ, MscL, AmtB).

## Native mass spectrometry

Membrane protein solutions (~5 µM) obtained upon exchange into detergent-containing ammonium acetate solutions (2‑3 µL) were loaded into a gold-coated, nano-electrospray ionization (nESI) capillary that was prepared in-house (outer diameter = 1.0 mm) and analyzed with Q-Exactive UHMR hybrid quadrupole-Orbitrap mass spectrometer (Thermo) using the following instrumental parameters: source temperature (100 °C), electrospray polarity (positive), capillary voltage (1.2 kV), injection flatapole (5 V), interflatapole lens (4 V), bent flatapole (2 V), transfer multipole (0 V), in-source trapping (50-100 V), higher-energy collisional dissociation (HCD) cell voltage (50-200 V), HCD cell pressure (9 x 10^-10^ bar), HCD cell gas (argon), noise level parameter (3), microscans (1‑10), and resolution (17,500 at m/z = 200).

## Average charge state calculation

The z_ave_ values were calculated using a previously established procedure:^[3]^ To calculate z_ave_, the spectral intensities of the protein charge states were plotted against the protein charge states (apo form). The data were fitted to a Gaussian function and the charge state (x-value) at maximum intensity was takes as the z_ave_ value.

## Relative intensities of apo form and protein-lipid complexes

The relative intensities of apo form and protein-lipid complexes were calculated using a previously established procedure:^[4]^ To monitor trends in delipidation, we calculated relative intensities of apo states and lipid-bound protein fractions and plotted the values against the detergent abbreviations. In every spectrum obtained from every protein-detergent combination, peak intensity values of apo states and lipid-bound protein fractions were extracted from the nMS data. The intensity values obtained from all peaks corresponding to the apo form and lipid-bound species within a spectrum were summed up to calculate the overall ion intensity. To determine the relative intensity of protein-lipid complexes within a spectrum, the overall intensity of protein-lipid complexes was divided by the overall ion intensity. To determine the relative intensity of the apo state within a spectrum, the overall intensity of the apo state was divided by the overall ion intensity. Relative intensity values of apo states and lipid-bound states were determined from three independent biological repeats (n=3), unless stated otherwise. Data were averaged and plotted against detergent abbreviations with standard error of the mean (±SEM).

# References

[1] C. Kirschbaum, K. Greis, S. Gewinner, W. Schöllkopf, G. Meijer, G. von Helden, K. Pagel, L. H. Urner, *ChempLusChem* **2024**, *89*, e202400340.

[2] L. H. Urner, A. Ariamajd, A. Weikum, *Chem. Sci.* **2022**, *13*, 10299-10307.

[3] L. H. Urner, I. Liko, H.-Y. Yen, K. K. Hoi, J. R. Bolla, J. Gault, F. G. Almeida, M.-P. Schweder, D. Shutin, S. Ehrmann, R. Haag, C. V. Robinson, K. Pagel, *Nat. Commun.* **2020**, *11*, 564.

[4] L. H. Urner, F. Fiorentino, D. Shutin, J. B. Sauer, M. T. Agasid, T. J. El-Baba, J. R. Bolla, P. J. Stansfeld, C. V. Robinson, *J. Am. Chem. Soc.* **2024**, *146*, 11025-11030.

[5] J. Gault, I. Liko, M. Landreh, D. Shutin, J. R. Bolla, D. Jefferies, M. Agasid, H.-Y. Yen, M. J. G. W. Ladds, D. P. Lane, S. Khalid, C. Mullen, P. N. Remes, R. Huguet, G. McAlister, M. Goodwin, R. Viner, J. E. P. Syka, C. V. Robinson, *Nat. Methods* **2020**, *17*, 505-508.

[6] R. A. Kramer, D. Zandwijken, M. R. Egmond, N. Dekker, *Eur. J. Biochem.* **2000**, *267*, 885-893.
